# Post-COVID-19 vaccination myocarditis: a prospective cohort study pre and post vaccination using cardiovascular magnetic resonance

**DOI:** 10.1186/s12968-023-00985-2

**Published:** 2023-12-07

**Authors:** Ming-Yen Ng, Cheuk Hang Tam, Yung Pok Lee, Ho Tung Ambrose Fong, Chun-Ka Wong, Wing Kei Carol Ng, Maegan Hon Yan Yeung, Wood-Hay Ian Ling, Sabrina Tsao, Eric Yuk Fai Wan, Vanessa Ferreira, Andrew T. Yan, Chung Wah Siu, Kai-Hang Yiu, Ivan Fan-Ngai Hung

**Affiliations:** 1 Department of Diagnostic Radiology, School of Clinical Medicine, Li Ka Shing Faculty of Medicine, The University of Hong Kong, Room 406, Block K, Queen Mary Hospital, Hong Kong SAR, China; 2 Department of Medical Imaging, HKU-Shenzhen Hospital, Shenzhen, China; 3 Department of Medicine, School of Clinical Medicine, Li Ka Shing Faculty of Medicine, The University of Hong Kong, Queen Mary Hospital, Hong Kong SAR, China; 4 Department of Radiology, Hong Kong Children’s Hospital, Hong Kong SAR, China; 5 Grantham Hospital, 125 Wong Chuk Hang Rd, Aberdeen, Hong Kong SAR, China; 6 Department of Paediatrics and Adolescent Medicine, School of Clinical Medicine, Li Ka Shing Faculty of Medicine, The University of Hong Kong, Hong Kong SAR, China; 7 Department of Family Medicine and Primary Care, The University of Hong Kong, Hong Kong SAR, China; 8 Department of Pharmacology and Pharmacy, The University of Hong Kong, Hong Kong SAR, China; 9 Division of Cardiovascular Medicine, Radcliffe Department of Medicine, Oxford BHF Centre of Research Excellence, Oxford Centre for Clinical Magnetic Resonance Research, NIHR Oxford Biomedical Research Centre, University of Oxford, Oxford, UK; 10 St. Michael’s Hospital, University of Toronto, Toronto, Canada

## Abstract

**Background:**

Concerns about COVID-19 vaccination induced myocarditis or subclinical myocarditis persists in some populations. Cardiac magnetic resonance imaging (CMR) has been used to detect signs of COVID-19 vaccination induced myocarditis. This study aims to: (i) characterise myocardial tissue, function, size before and after COVID-19 vaccination, (ii) determine if there is imaging evidence of subclinical myocardial inflammation or injury after vaccination using CMR.

**Methods:**

Subjects aged ≥ 12yrs old without prior COVID-19 or COVID-19 vaccination underwent two CMR examinations: first, ≤ 14 days before the first COVID-19 vaccination and a second time ≤ 14 days after the second COVID-19 vaccination. Biventricular indices, ejection fraction (EF), global longitudinal strain (GLS), late gadolinium enhancement (LGE), left ventricular (LV) myocardial native T1, T2, extracellular volume (ECV) quantification, lactate dehydrogenase (LDH), white cell count (WCC), C-reactive protein (CRP), NT-proBNP, troponin-T, electrocardiogram (ECG), and 6-min walk test were assessed in a blinded fashion.

**Results:**

67 subjects were included. First and second CMR examinations were performed a median of 4 days before the first vaccination (interquartile range 1–8 days) and 5 days (interquartile range 3–6 days) after the second vaccination respectively. No significant change in global native T1, T2, ECV, LV EF, right ventricular EF, LV GLS, LGE, ECG, LDH, troponin-T and 6-min walk test was demonstrated after COVID-19 vaccination. There was a significant WCC decrease (6.51 ± 1.49 vs 5.98 ± 1.65, p = 0.003) and CRP increase (0.40 ± 0.22 vs 0.50 ± 0.29, p = 0.004).

**Conclusion:**

This study found no imaging, biochemical or ECG evidence of myocardial injury or inflammation post COVID-19 vaccination, thus providing some reassurance that COVID-19 vaccinations do not typically cause subclinical myocarditis.

## Introduction

Novel messenger ribonucleic acid (mRNA) vaccinations for coronavirus disease 2019 (COVID-19) has been shown to very infrequently cause myocarditis [1–3] which in the most severe situations has led to death or heart failure [4]. Patients with COVID-19 vaccination induced myocarditis almost all present with chest pain and approximately 30% may have shortness of breath and fever [5, 6]. Although this complication is rare (21.3 to 33.3 cases per million doses) [7, 8], it has raised significant concern in the population and medical community resulting in refusals to receive COVID-19 vaccination. These studies found that myocarditis occurred usually within the first 7 days of vaccination and more commonly after the second dose.

The mechanism leading to the complication of vaccine-induced myocarditis is unknown. However, cardiac magnetic resonance imaging (CMR) has taken on a prominent role in identifying COVID-19 related myocarditis [9, 10] and vaccine related myocarditis [11, 12]. CMR has unique imaging tools allowing the identification of myocardial oedema and fibrosis, and thus provides a non-invasive assessment of the myocardial tissue, which, in the past, would have required biopsy. The imaging diagnosis of myocarditis using CMR is one using the updated Lake Louise criteria (LLC) [13], which requires fulfilment of at least one T2-based oedema imaging criterion (ie. T2 mapping or T2-weighted imaging), and a T1-based imaging criterion (T1 mapping, extracellular volume (ECV) or late gadolinium enhancement (LGE)), to support the diagnosis. However, one concern with using CMR to diagnose vaccine-induced myocarditis has been the lack of CMR examination prior to the episode, leading to uncertainty whether some of the CMR changes like elevated native T1 or LGE in the myocardium were pre-existing, and therefore leading to false attribution of the myocarditis findings as vaccine-induced.

In order to better understand whether there are myocardial changes that occur post vaccination, we undertook this prospective cohort study of subjects undergoing COVID-19 vaccination pre- and post-vaccination to determine if COVID-19 vaccination induces subclinical myocardial inflammation.

## Methods

Research ethics approval was obtained from the Hong Kong West Cluster Institutional Review Board. This prospective cohort study is registered on ClinicalTrials.gov (No. NCT05184114).

Participants were invited into our study through online media and physical posters. Recruitment occurred from September 2021 to February 2022. At the time of this study’s initiation, Hong Kong had a well-documented extremely small number of COVID-19 infections in the population (ie. < 15,000 COVID-19 cases out of a population of > 7 million people by 1st February 2022) [14]. Inclusion criteria were (i) participants > 12 years old and (2) no prior COVID-19 vaccination or COVID-19 infection. Exclusion criteria were (1) history of cardiac disease i.e. myocardial infarction, myocarditis, heart failure, (ii) presence of pacemakers or implantable cardiac defibrillators, (iii) any contraindication for CMR testing, (iv) Renal impairment with estimated glomerular filtration rate (eGFR) < 45 ml/min/1.73m^2^, (v) Limited life expectancy < 1 year, (e.g. cancer or liver failure), (vi) refusal or inability to sign an informed consent, (vii) Suboptimal image quality due to artefacts. All participants provided written informed consent. Participants underwent 2 CMR studies within a designated tight time window. The first CMR scan occurred ≤ 14 days prior to the first vaccination, and second scan was performed ≤ 14 days after the second vaccination. The 2nd scan’s time frame after the 2nd vaccination was based on previous publications which indicated that the onset of myocarditis symptoms usually happened within 14 days after second dose inoculation [1].

Participants enrolled into the study undertook over-the-counter COVID-19 antibody testing and blood tests at the time of 1st and 2nd CMR scans for haematocrit, lactate dehydrogenase (LDH), white cell count (WCC), C-reactive protein (CRP), N-terminal pro-brain natriuretic peptide (NT-proBNP) and high sensitivity troponin-T. An electrocardiogram and 6- minute walk test were also performed on the same day as the CMR scans. Signs and symptoms after vaccination were recorded at the time of the second CMR examination.

### CMR examination

All scans were performed on a GE 1.5 T MR scanner. Standard multiplanar cine imaging for cardiac function assessment in the 2, 3 and 4 chamber views were obtained. T2 short tau inversion recovery (STIR) were acquired as a whole left ventricular short axis stack. Pre and post-contrast T1 modified Look-Locker Inversion Recovery (MOLLI) sequences and T2 mapping sequences were acquired in the basal, mid-ventricular and apical short axis positions. Pre-contrast T1 MOLLI sequence used a 5(3)3 sampling scheme and the post-contrast T1 MOLLI sequence used a 4(1)3(1)2 sampling scheme. Pre-contrast T1-map scanning parameters were as follows: time to echo (TE) 1.5 ms, time to repetition (TR) 3.5 ms, flip angle 35 degrees, field of view (FOV) 40 cm x 40 cm, spatial resolution 1.6 mm × 1.6 mm, slice thickness 10 mm. T2 mapping images of the basal, mid-ventricular and apical short axis oblique slices were also acquired in the same position as the T1 mapping images. The sequence parameters were as follows: fast spin echo, TE 10.5 ms, TR 1017 ms, flip angle 90 degrees, FOV 40 cm x 40 cm, spatial resolution 1.8 mm × 1.8 mm, slice thickness 10 mm. For post-contrast T1 MOLLI maps, images were obtained 15 min after intravenous gadolinium-based contrast agent Dotarem (0.1 mmol/kg) was administered. A TI scout was acquired to identify the optimum nulling time. LGE was initiated 5 min post contrast.

### CMR analysis

Image analysis and interpretation was done blindly by 2 dedicated CMR analysts and reviewed by an experienced CMR reader (MYN & WKCN) using cvi42 (Circle Cardiovascular Imaging, Calgary, Canada). Left ventricular (LV) and right ventricular (RV) indices were assessed by its end diastolic volume (EDV) indexed by body surface area, corrected end systolic volume (ESV) indexed by body surface area, cardiac index (CI), ejection fraction (EF), and global longitudinal strain (GLS). Volumes and ejection fraction were obtained by contouring the ventricular endocardial and epicardial surfaces in the end-diastolic and end-systolic phases. GLS was obtained by contouring the LV endocardial and epicardial surfaces on the 2-chamber and 4-chamber cine in end-diastole and calculated using the cvi42 software as previously described [15].

Global native T1, T2 and ECV was determined by contouring the entire myocardium on the mid-ventricular slice, while segmental values were obtained by contouring regions of interest in the AHA 16 segments. Segments with observable artefacts and significant movement between phases were excluded for segmental analysis. ECV quantification was calculated as previously described [16], using pre-contrast native T1 MOLLI and post-contrast T1 MOLLI of both the myocardium and blood pool, as well as the haematocrit acquired at the time of CMR examination. LGE images were assessed visually for presence or absence of infarct or fibrosis.

A CMR imaging diagnosis of non-ischaemic myocardial inflammation was based on the revised Lake Louise criteria (LLC) [13].

### Inter-observer/intra-observer variability

20 participants were randomly selected for inter-observer variability assessment of global native T1, global native T2, ECV and GLS measurements. This was done by two independent readers (CHT & YPL). Cases were contoured more than 4 weeks apart from initial contouring.

### Sample size calculation

Based on calculations of previous native T1 MOLLI and native T2 measurements the following one-sample size calculations were made using an alpha of 0.05 and power of 0.90. Using a mean native T1 value of 1050 ms to identify a difference of 25 ms, standard deviation of 60 ms, a sample size of 63 patients would be required. For native T2, using a mean of 48 ms to identify a difference of 5 ms with a standard deviation of 10 ms, a sample size of 44 patients would be required. Therefore, based on these calculations, we would aim to recruit a final cohort of at least 63 patients.

### Statistical analysis

The Shapiro–Wilk test was used to determine the normal distribution of variables. Paired t-test was used to compare the means of normally distributed continuous data and Wlicoxon signed-rank test was used for non-normally distributed continuous data before and after vaccination. McNemar’s test was performed for categorical variables before and after vaccination. p-value < 0.05 was deemed statistically significant. Statistical analysis was performed on Stata version 14.2 (Stata Corporation, College Station, Texas, United States).

Fulfilment of the updated LLC required demonstration of significant changes between the 1st (pre-vaccination) and 2nd CMR scans (post-vaccination after two doses) in CMR parameters for both a T2-based parameter for myocardial oedema (T2-mapping) and a T1-based parameter (T1-mapping or ECV or LGE). Ancillary diagnostic features suggestive of myocarditis, such as pericardial effusion and systolic LV function, were also analysed for post-vaccination changes [13].

For inter and intra-observer variability assessment, intraclass correlation coefficient (ICC) values for inter-observer reliability were performed.

## Results

**Fig. 1:**
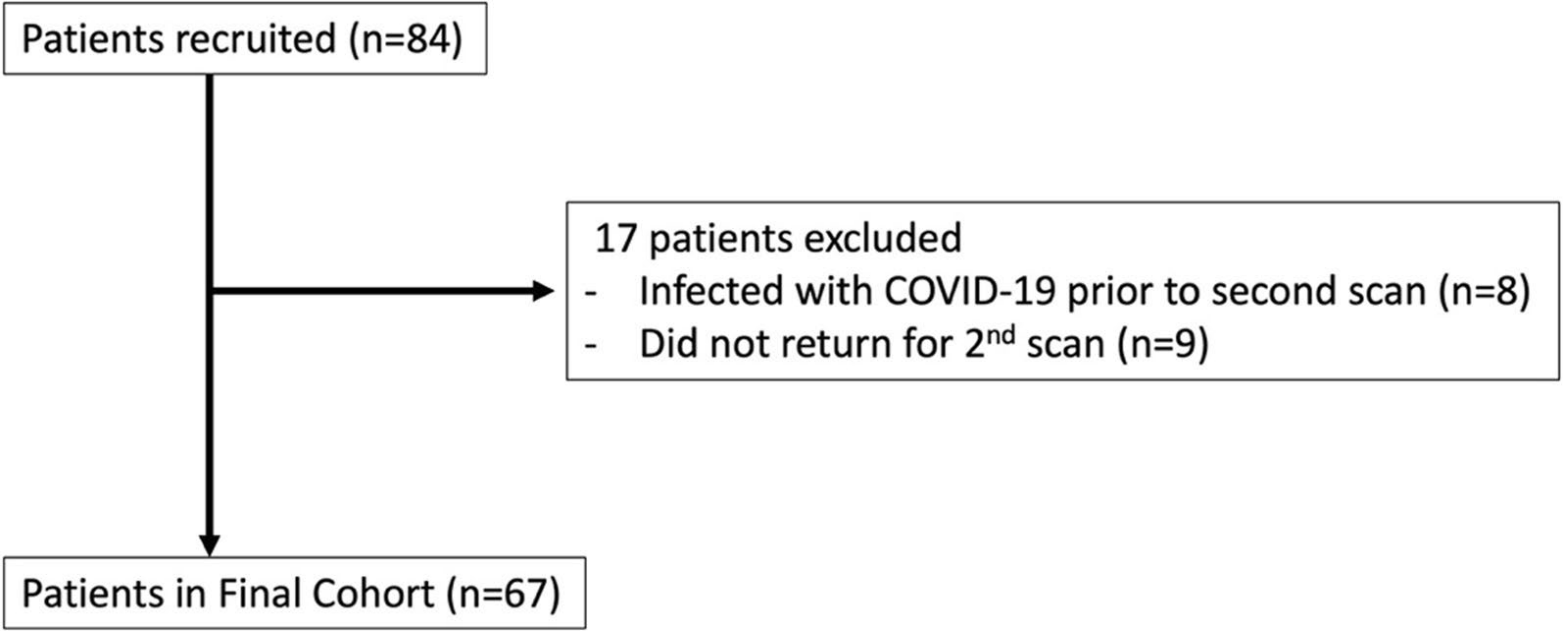
CONSORT diagram of the study recruitment

84 subjects were recruited. 17 were excluded. 8 were excluded for acquiring COVID-19 infection during the interval and 9 did not return for the second scan (see Fig. 1 for CONSORT diagram). A total of 67 participants (30 males (44.8%), median age 30 years, range 12–75 years) completed the entire study. 2 CMR scans were done, one before and one after vaccination. The 1st CMR scan was performed a median of 4 days (interquartile range 1–8 days) before. The 2^nd^ CMR scan was performed a median of 5 days (interquartile range 3–6 days) after the 2^nd^ vaccination.

### Demographics

Participant demographics are illustrated in Table 1. Cardiovascular risk factors were present in a small minority of participants of which hypertension and hyperlipidaemia (4 participants, 6.0% for both) were the most common. Statins were the most common medication utilised but in a small minority of participants (4 participants, 6.0%). Briefly, 88.1% of participants had the BNT162b2 (Comirnaty, BioNtech, Mainz, Germany) vaccine and the remainder had the CoronaVac vaccine (Sinovac Biotech Ltd., Beijing, China). No participant received different vaccines for the 1^st^ and 2nd doses. 60 out of 67 participants (89.6%) had both injections in their left arm. 5 subjects had both injections in their right arm. 2 participants received injections in their left thigh for both doses which had previously been suggested to reduce the risk of COVID-19 vaccination induced myocarditis[17]. The most common symptoms after the 2nd vaccination were myalgia (37 participants, 55.2%) and fatigue (33 participants, 49.3%). Chest pain (15 participants, 22.4%) and shortness of breath (11 participants, 16.4%) occurred in a proportion of participants.

**Table 1:** Participant demographics

	Participant cohort (n = 67)
Male	30 (44.8%)
Age (yrs)	30 (25–41)
BNT162b2 Vaccine	59 (88.1%)
CoronaVac Vaccine	8 (11.9%)
No. of Days between 1st vaccination and 1st CMR Scan Examination (days)	4 (1–8)
No. of Days between 2nd vaccination and 2nd CMR Scan Examination (days)	4.7 $\pm$ 2.4
Days between 1st and 2nd Vaccine Dose (days)	24 (21–28)
1^st^ Vaccination Injected into the Left Arm	60 (89.6%)
1^st^ Vaccination Injected into the Right Arm	5 (7.5%)
1^st^ Vaccination Injected into the Left Thigh	2 (3.0%)
2^nd^ Vaccination Injected into the Left Arm	60 (89.6%)
2^nd^ Vaccination Injected into the Right Arm	5 (7.5%)
2^nd^ Vaccination Injected into the Left Thigh	2 (3.0%)
*Symptoms after 2nd COVID-19 Vaccination Dose*	
Chest pain	15 (22.4%)
Vomiting	0 (0.0%)
Nausea	4 (6.0%)
Pyrexia (≥ 38$^{{^{ \circ } }}$C)	6 (9.0%)
Myalgia	37 (55.2%)
Fatigue	33 (49.3%)
Shortness of breath	11 (16.4%)
Palpitations	7 (10.4%)
*Cardiac risk factors & Co-Morbidities*	
Hypertension	4 (6.0%)
Hyperlipidaemia	4 (6.0%)
Obesity	1 (1.5%)
Smoking	3 (4.5%)
Diabetes Mellites Type 2	2 (3.0%)
Previous CABG	0 (0.0%)
Previous coronary stent	0 (0.0%)
Previous history of cancer	0 (0.0%)
*Drugs*	
ACEI/ ARB	1 (1.5%)
Beta-blockers	2 (3.0%)
Calcium channel blockers	2 (3.0%)
Diuretics	0 (0.0%)
Clopidogrel	0 (0.0%)
Aspirin	1 (1.5%)
Anti-diabetic	0 (0.0%)
Statin	4 (6.0%)

Variables are presented as number of participants and percentage in brackets for categorial data. For continuous variables, median with interquartile range or mean with standard deviation are displayed if the variables are normally distributed or not normally distributed

After the 2nd vaccination (see Table 2), there was a statistically significant decrease in WCC (6.51$\pm$1.49 vs 5.98 $\pm$ 1.65, p = 0.003) and increase in CRP (0.40 $\pm$ 0.22 vs 0.50 $\pm$ 0.29, p = 0.004). Haemoglobin, haematocrit, eGFR, LDH, troponin-T and NT-proBNP showed no significant change. COVID-19 antibodies were absent in all participants prior to vaccination. After the 2nd vaccination, COVID-19 antibodies were present in 82.1% of participants. ECGs showed no change pre and post vaccination in terms of development of new ST/ T-wave changes, QT interval, rhythm, QRS complexes or q-wave development. One patient had Q-waves in the inferior leads pre and post vaccination.

**Table 2:** Blood Results Before and After 2 Vaccination Doses (n = 67)

Participant cohort (n = 67)	Before 1st vaccine dose	After 2nd vaccine dose	p-value
Hb (g/dL)	13.0 $\pm$ 1.6	13.8 $\pm$ 1.6	0.225
Hct	0.42 $\pm$ 0.04	0.42$\pm$0.04	0.741
eGFR (ml/min/1.73m2)	81.3 $\pm$19.5	88.0 $\pm$ 6.6	0.203
WCC (10^9^/L)	6.51 $\pm$1.49	5.98 $\pm$ 1.65	0.003*
CRP (mg/L)	0.40 $\pm$ 0.22	0.50 $\pm$ 0.29	0.004*
LDH (units/L)	199.8 $\pm$39.0	205.3 $\pm$ 53.1	0.454
Troponin-T (ng/L)	5 (5–6.2)	5 (5–5.9)	0.311
NT-pro-BNP (pg/ml)	35.6 $\pm$ 23.3	31.4 $\pm$ 27.1	0.212
6 min walk test (m)	386.7 $\pm$ 69.4	387.0 $\pm$ 42.3	0.963
COVID-19 antibodies present (%)	0.0	82.1	< 0.001*

Results are presented as mean and standard deviation

*Hb* haemoglobin*, Hct* Haematocrit*, eGF* Restimated glomerular filtration rate*, WCC* white cell count*, CRPC-*reactive protein*, LDH* lactate dehydrogenase*, NT-proBNP N-*terminal pro hormone brain natriuretic peptide

^*^ = *p* < *0.05*

There was no significant difference in the 6- minute walk test before and after vaccination.

### CMR findings

**Fig. 2:**
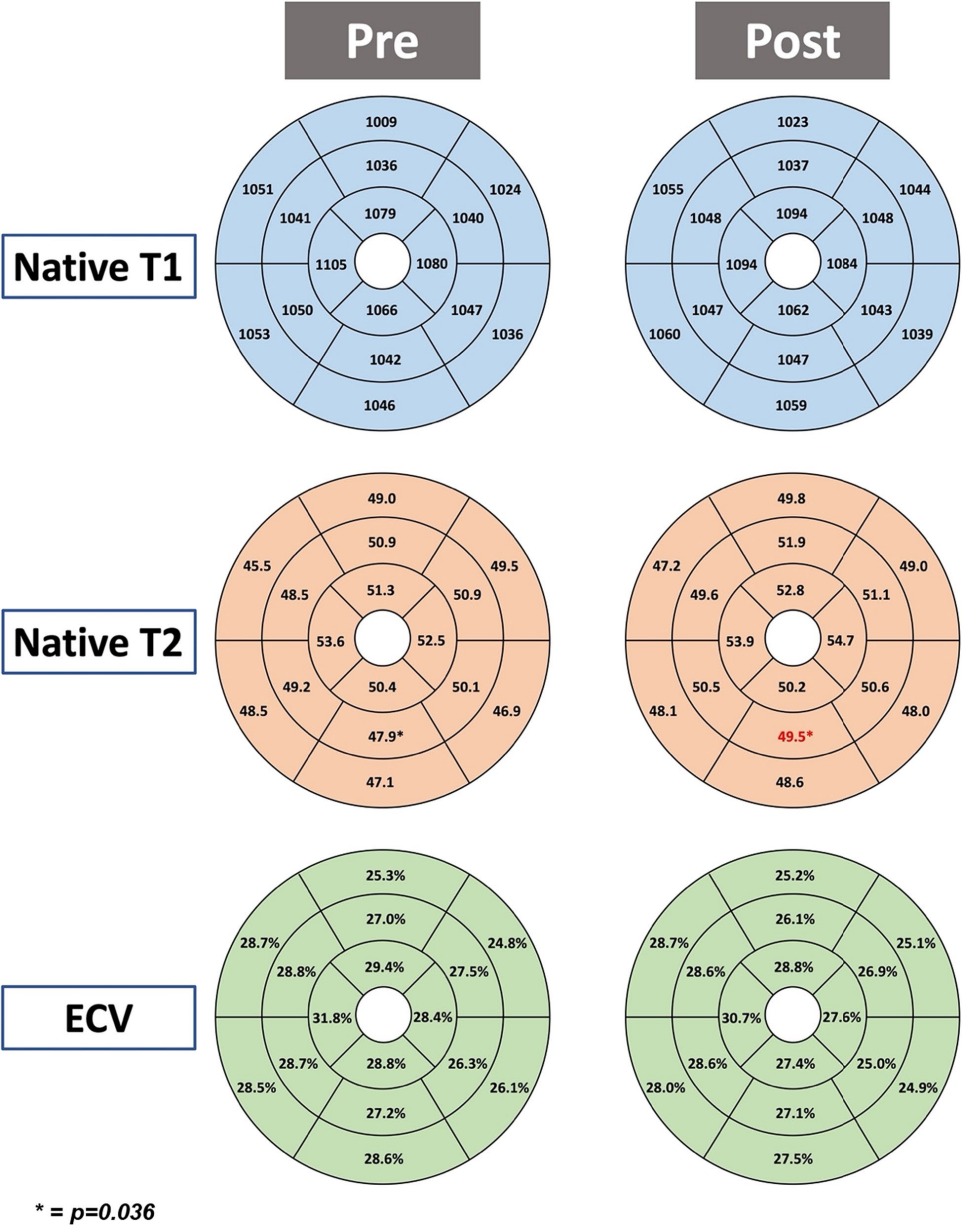
Mean native T1, native T2 and extracellular volume (ECV) by American Heart Association segments of the left ventricle pre and post vaccination (n = 67). Native T1 and native T2 values are in milliseconds

**Fig. 3:**
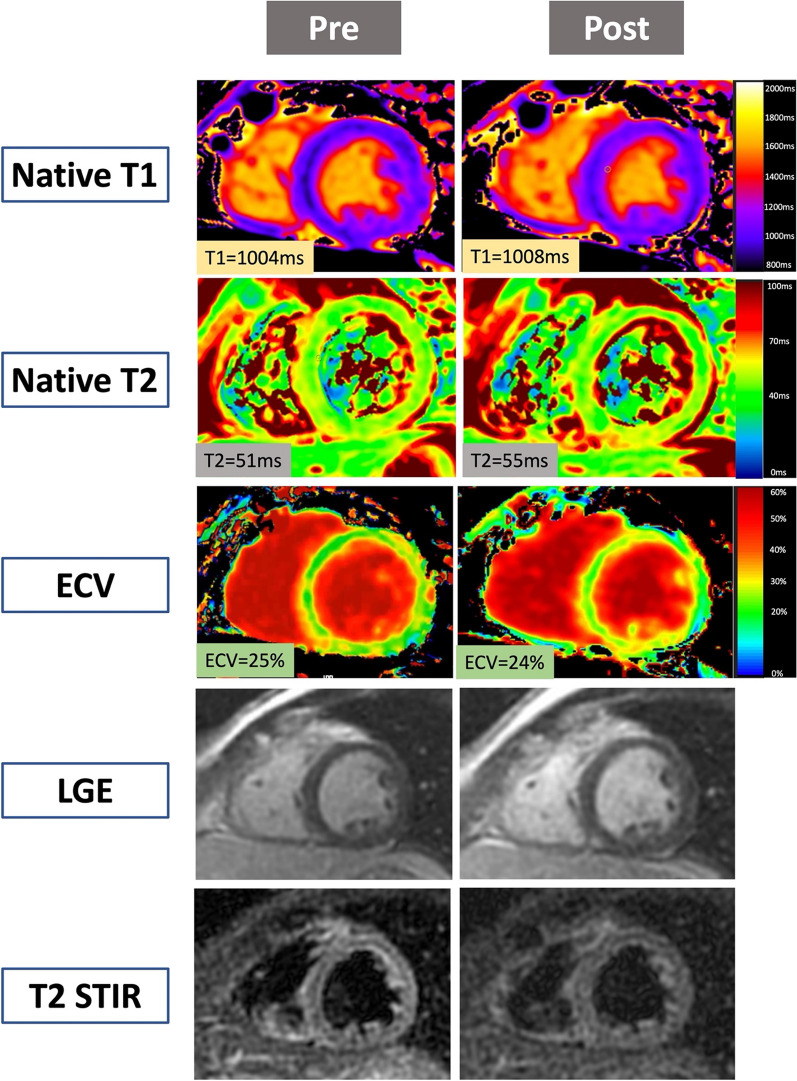
36 yr old male with pre and post COVID-19 vaccination CMR examinations. Native T1 map, native T2 map, extracellular volume (ECV) map, late gadolinium enhancement (LGE) and T2 short tau inversion recovery (STIR) images are displayed below

CMR results before and after two doses of COVID-19 vaccinations for all participants in our cohort (n = 67) are shown in Table 3. CMR left ventricular (LV), right ventricular and atrial parameters did not show any significant change between the 1st and 2nd scans. There was no significant difference in LV GLS (p = 0.881) after the 2nd vaccination dose, and no pericardial effusion was visible. Global native T1, native T2 and ECV showed no significant change (see Fig. 2 and 3). When assessing the participants’ myocardium on an American Heart Association segmental level, there was no significant change amongst the segments for native T1 and ECV. However, for native T2, there was a slight increase in native T2 values in segment 10 (p = 0.036) which represents the mid-ventricular inferior wall. Only 1 out of the 67 participants in our cohort demonstrated minor non-specific LGE at the mid-ventricular anterior wall on both 1st and 2nd CMR scans. No new LGE or high T2 signal changes in the myocardium or pericardium were demonstrated on the LGE or T2 STIR images in any of the subjects. No participant fulfilled the updated LLC for myocarditis.

**Table 3:** Cardiac Magnetic Resonance Results Before and After 2 Vaccination Doses (n = 67)

	Before 1st vaccine dose	After 2nd vaccine dose	p-value
LV EDV Indexed (ml/m^2^)	81 $\pm$ 12	81 $\pm$ 12	0.798
LV ESV Indexed (ml/m^2^)	35 $\pm$ 7.0	35 $\pm$ 8.2	0.854
LV EF (%)	58 $\pm$ 5.0	57 $\pm$ 5.3	0.088
LV Mass Indexed (g/m^2^)	46 $\pm$ 8.4	45 $\pm$ 8.6	0.094
Cardiac Index (L/min/m^2^)	3.3 $\pm$ 0.50	3.2 $\pm$ 0.6	0.431
Global Longitudinal Strain (%)	17 $\pm$ 2.0	17 $\pm$1.8	0.881
RV EDV Indexed (ml/m^2^)	87 $\pm$ 18	87 $\pm$ 15	0.987
RV ESV Indexed (ml/m^2^)	40 $\pm$9.2	41 $\pm$ 9.7	0.627
RV EF (%)	54 $\pm$ 5.5	54 $\pm$ 5.7	0.412
LA Area Corrected (cm^2^/m^2^)	13 $\pm$ 1.8	12 $\pm$ 1.8	0.088
RA Area Corrected (cm^2^/m^2^)	12 $\pm$ 2.2	12 $\pm$ 2.6	0.329
Pericardial Effusion (n, %)	1 (1.5%)	1 (1.5%)	1.00
Global Native T1 Myocardium (msec)	1045$\pm$50	1055 $\pm$ 55	0.303
Global Native T2 Myocardium (msec)	49.5 $\pm$ 4.2	50.5 $\pm$ 4.4	0.191
ECV Myocardium (%)	27.9 $\pm$ 3.8	27.8 $\pm$ 4.4	0.901
LGE Present	1 (1.5%)	1 (1.5%)	1.00
T2 STIR Abnormalities	0	0	1.00
Lake Louise Criteria Fulfilled (%)	0%	0%	1.00

Results are presented as mean and standard deviation

*LV left ventricle, EDV corrected end diastolic volume, ESV corrected end systolic volume, EF ejection fraction, RV right ventricle, ECV extracellular volume, LGE late gadolinium enhancement, STIR short tau inversion recovery*

In patients with chest pain, palpitations or shortness of breath before and after COVID-19 vaccination, there was no significant change in native T1, T2 or ECV (p > 0.05) (see Table 4).

**Table 4:** Comparing T1, T2 and extracellular volume in patients with cardiac symptoms before and after COVID-19 vaccination

	Before 1st Vaccine Dose	After 2nd Vaccine Dose	p-value
Patients with chest pain (n = 15)			
Global Native T1 Myocardium (msec)	1080 (1005–1101)	1039 (1007–1081)	0.520
Global Native T2 Myocardium (msec)	48.5 (44.5–51.2)	48.8 (46.5–52.1)	0.756
ECV Myocardium (%)	28.4 (26.4–32.3)	27.0 (24.6–31.1)	0.254
Patients with palpitations (n = 7)			
Global Native T1 Myocardium (msec)	1086 (1044–1099)	1088 (1039–1139)	0.749
Global Native T2 Myocardium (msec)	53.1 (46.2–57.6)	46.7 (45.9–53.2)	0.338
ECV myocardium (%)	28.3 (26.4–31.9)	27.0 (24.6–32.5)	0.565
Patients with SOB (n = 11)			
Global Native T1 Myocardium (msec)	1022 (979–1090)	1049 (998–1081)	0.533
Global Native T2 Myocardium (msec)	49.3 (46.6–53.1)	48.2 (46.5–52.7)	0.670
ECV Myocardium (%)	26.3 (22.3–28.6)	24.6 (22.6–28.7)	0.922

Variables are presented as median with interquartile range

*ECV* extracellular volume*; SOB* shortness of breath

Inter-observer reliability ranged from good to excellent based on ICC values of global native T1 (0.966), global native T2 (0.806), and ECV values (0.939).

## Discussion

Our prospective cohort study was specifically designed to investigate whether COVID-19 vaccinations induce subclinical myocardial inflammation in adolescents and adults with no significant medical history using CMR imaging pre and post vaccination [18, 19]. None of our participants met the updated LLC for diagnosis of myocardial inflammation or demonstrated significant changes in CMR parameters of cardiac function after two doses of COVID-19 vaccinations. In addition, we found no significant change in troponin-T, NT-proBNP, LDH and ECG. Furthermore, none of the sub-group of patients with chest pain, shortness of breath or palpitations subsequent to COVID-19 vaccination developed elevations in native T1, T2 or ECV. This result fills an important knowledge gap with prospective CMR evidence, demonstrating that the COVID-19 vaccination did not induce subclinical myocardial inflammation in individuals included in this study, who had no significant cardiac history.

This supplements the existing literature on COVID-19 vaccine-induced myocarditis. Current literature indicates that the risk of myocarditis post vaccination is low (0.00002% to 0.007%)[7, 20] but whether there is underlying subclinical myocarditis/ inflammation is uncertain. In this study, we excluded subjects with previous COVID-19 infections using screening of COVID-19 antibodies prior to enrolment. Furthermore, at the time of this study’s initiation, Hong Kong had an extremely small number of COVID-19 infections in the population (ie. < 15,000 COVID-19 cases out of a population of > 7 million people by 1^st^ February 2022 throughout the pandemic) [14]. Thus, this was a unique population to assess the effect of COVID-19 vaccinations in subjects without previous COVID-19 infection and naïve to COVID-19 vaccination.

In our cohort, we also had subjects that developed symptoms similar to myocarditis. However, none of these patients fulfilled LLC criteria for myocarditis. This suggests that in addition to clinical symptoms other evidence of myocardial inflammation such as elevated troponin levels and ECG changes are required before considering patients for CMR examination. In this cohort, none of the patients demonstrated elevated troponin levels or significant ECG changes to raise suspicion of myocardial inflammation. Previous studies indicate that > 50% of patients had elevated troponin levels as well as ECG changes [6, 7].

A recently published study by Nakahara et al. [21], used 18-fluorodeoxyglucose (^18^F-FDG) positron emission tomography- computed tomography (PET-CT) scans to assess asymptomatic patients pre and post vaccination. Their study showed increased myocardial inflammation post vaccination. These results are discordant with our study but the differences can be explained by the subject population of the two studies and the imaging techniques used. Nakahara et al., have a population of patients that underwent PET-CT of which roughly half had cancer. Their chemotherapy and radiotherapy regimens are unknown, thus the influence of these treatments on their findings cannot be determined. Crucially, although COVID-19 infection was an exclusion criteria in their study, undetected COVID-19 could still be a confounder with the timing of the non-vaccination group when COVID-19 infections were substantially lower (1^st^ November 2020 to 16^th^ February 2021 had a maximum of > 7,000 COVID-19 cases per day) whilst the vaccination period occurring when the incidence of COVID-19 was markedly higher (17^th^ March 2021 to 31^st^ March 2022 with a maximum of > 100,000 cases per day) [14]. COVID-19 infection is well established to more frequently cause myocardial inflammation and more so than COVID-19 vaccinations [22]. Thus active COVID-19 screening was performed in our study using questionnaires and blood tests at the time of pre and post vaccination CMR scanning but active screening was not stated in Nakahara’s study. During both study periods Japan had significantly more COVID-19 infections than Hong Kong. Japan had > 2.8 million cumulative COVID-19 infections with > 80,000 cases per day on 1st February 2022 whilst in Hong Kong there were < 15,000 cumulative cases on 1st February 2022 [14]. This was also a retrospective study without measurement of cardiac enzymes/ inflammatory markers, ECG or cardiac function to corroborate the PET-CT findings so whether the increased myocardial activity is due to underlying inflammation or another confounder like metabolism or previous chemotherapy agents is difficult to determine. We measured cardiac enzyme/ inflammatory markers, ECG and cardiac function before and after vaccination. Our study’s cardiac enzymes/ inflammatory markers, ECG and cardiac function corroborate our findings. In addition, our population were healthy and underwent second CMR scans within 14 days which is the peak period expected for myocarditis [1] whilst Nakahara’s study had patients with second scans from 1 day to > 180 days after vaccination. In addition, ^18^F-FDG PET-CT is not a good tracer for measuring myocardial activity[23] and not recommended for assessing myocardial inflammation [24]. Furthermore, a special low carbohydrate and high fat diet is recommended if myocardial activity is to be measured [23]. This diet was not incorporated routinely in Nakahara’s study. The editorial accompanying Nakahara et al.’s paper highlights other limitations with ^18^F-FDG PET-CT for assessing myocardial inflammation [23]. Alternatively CMR, which was used in our study, is a recommended test for assessing myocardial inflammation[24] and has been shown to have a high diagnostic accuracy for assessment of myocardial inflammation (sensitivity 87.5%; specificity 96.2%) [25] and has multiple tools to help identify myocarditis including T1/ T2 mapping, LGE and T2 weighted fat suppression sequences for diagnosis [25].

COVID-19 vaccination has a wealth of data showing that it is effective and safe [22, 26, 27]. The incidence of COVID-19 induced myocarditis is low with 21.3 to 33.3 cases per million doses [7, 8] and if myocarditis does occur, it rarely leads to death or heart failure [4]. Thus our study adds mechanistic information that subclinical myocardial inflammation does not usually occur in subjects post COVID-19 vaccination and would partly explain why COVID-19 vaccine induced myocarditis is uncommon.

We previously showed that a high blood mRNA vaccine level can cause myopericarditis. In patients who experienced myocarditis post COVD-19 vaccination, we postulated that this could have occurred due to rapid movement of the vaccine via the lymphatic system and thus changing the vaccination site may reduce the risk [17]. Thus it was suggested that vaccinations delivered in the thigh may provide a reduced risk of myocarditis due to the enhanced uptake by macrophages and dendritic cells at the para-aortic, inguinal and iliac lymph nodes [17]. In our study, 97% of subjects received their vaccinations in the arm. With only two subjects having injections in their thigh, it is hard to draw conclusions from the current data.

### Strengths & Limitations

The strength of our study included CMR examinations being performed within 2 weeks of the 2nd COVID-19 vaccination as previous studies have shown that COVID-19 vaccination related myocarditis commonly occurs in this period and usually after the second vaccination [7, 8, 20]. Furthermore, we included adolescents since this group has been shown to be higher risk of COVID-19 vaccine related myocarditis [7].

Our study has several limitations. Firstly, our study represents a predominantly Chinese population and therefore results may not be generalisable to different ethnic groups. Secondly, the study has a relatively small sample size and maybe under powered to detect smaller changes due to COVID-19 vaccinations. Thirdly, the cohort is a healthy population with no history of cardiac disease and no previous COVID-19 infection. However, up to 22% of our subjects developed symptoms such as chest pain, shortness of breath and pyrexia which is similar to patients that developed myocarditis induced by COVID-19 vaccination. Furthermore longer term changes is unknown but long-term follow-up of these participants would be useful in delineating long-term cardiac sequalae of COVID-19 vaccination in these participants.

## Conclusion

In this prospective cohort study, COVID-19 vaccination did not induce any CMR imaging, blood marker or ECG evidence of myocardial inflammation in individuals with no significant cardiac history. None of our subjects met the updated LLC for diagnosis of myocarditis or demonstrated significant changes in CMR parameters of cardiac function after two doses of COVID-19 vaccinations. Even in the sub-group of subjects developing chest pain, shortness of breath and palpitations post vaccination did not show increase in native T1, T2 or ECV. These findings can hopefully contribute constructively to the discussion of vaccine hesitancy.

## Data Availability

The datasets used and/or analyzed during the current study are available from the corresponding author on reasonable request.

## Abbreviations

^18^F-FDG: 18 (^18^F) fluorodeoxyglucose
AHA: American Heart Association
CI: Cardiac index
CMR: Cardiovascular magnetic resonance
COVID-19: Coronavirus disease 2019
CRP: C-reactive protein
ECG: Electrocardiogram
ECV: Extracellular Volume
EDV: End-diastolic volume
EF: Ejection fraction
ESV: End-systolic volume
eGFR: Estimated glomerular filtration rate
FOV: Field of View
GLS: Global longitudinal strain
ICC: Intraclass correlation coefficient
LDH: Lactate dehydrogenase
LGE: Late gadolinium enhancement
LLC: Lake Louise criteria
LV: Left Ventricle
MOLLI: Modified Look-Locker Inversion Recovery
NT-proBNP: N-terminal pro-brain natriuretic peptide
PET-CT: Positron emission tomography- computed tomography
RV: Right ventricle
STIR: Short tau inversion recovery
TE: Time to echo
TR: Time to repetition
WCC: White cell count

## References

[1] Mevorach D (2021). Myocarditis after BNT162b2 mRNA vaccine against Covid-19 in Israel. N Engl J Med.

[2] Oster ME (2022). Myocarditis cases reported after mRNA-based COVID-19 vaccination in the US from december 2020 to august 2021. JAMA.

[3] Lai FTT (2022). Carditis after COVID-19 vaccination with a messenger RNA vaccine and an inactivated virus vaccine: a case-control study. Ann Intern Med.

[4] Tschope C (2019). Management of myocarditis-related cardiomyopathy in adults. Circ Res.

[5] Truong DT (2022). Clinically suspected myocarditis temporally related to COVID-19 vaccination in adolescents and young adults: Suspected myocarditis after COVID-19 vaccination. Circulation.

[6] Chua GT (2021). Epidemiology of acute myocarditis/pericarditis in Hong Kong adolescents following comirnaty vaccination. Clin Infect Dis.

[7] Witberg G (2021). Myocarditis after Covid-19 vaccination in a large health care organization. N Engl J Med.

[8] Ling RR (2022). Myopericarditis following COVID-19 vaccination and non-COVID-19 vaccination: a systematic review and meta-analysis. Lancet Respir Med.

[9] Kotecha T (2021). Patterns of myocardial injury in recovered troponin-positive COVID-19 patients assessed by cardiovascular magnetic resonance. Eur Heart J.

[10] Ng MY (2020). Patients recovered from COVID-19 show ongoing subclinical myocarditis as revealed by cardiac magnetic resonance imaging. JACC Cardiovasc Imaging.

[11] Shiyovich A (2022). Myocarditis following COVID-19 vaccination: magnetic resonance imaging study. Eur Heart J Cardiovasc Imaging.

[12] Fronza M (2022). Myocardial injury pattern at MRI in COVID-19 vaccine-associated myocarditis. Radiology.

[13] Ferreira VM (2018). Cardiovascular magnetic resonance in nonischemic myocardial inflammation: expert recommendations. J Am Coll Cardiol.

[14] Dong E, Du H, Gardner L (2020). An interactive web-based dashboard to track COVID-19 in real time. Lancet Infect Dis.

[15] Ng M-Y (2023). Diagnostic accuracy of cardiovascular magnetic resonance strain analysis and atrial size to identify heart failure with preserved ejection fraction. Eur Heart J Open.

[16] Messroghli DR (2017). Clinical recommendations for cardiovascular magnetic resonance mapping of T1, T2, T2* and extracellular volume: a consensus statement by the society for cardiovascular magnetic resonance (SCMR) endorsed by the European association for cardiovascular imaging (EACVI). J Cardiovasc Magn Reson.

[17] Li C (2021). Intravenous injection of coronavirus disease 2019 (COVID-19) mRNA vaccine can induce acute myopericarditis in mouse model. Clin Infect Dis.

[18] Fatima M (2022). Development of myocarditis and pericarditis after COVID-19 vaccination in adult population: a systematic review. Ann Med Surg (Lond).

[19] Samimisedeh P (2022). Cardiac MRI Findings in COVID-19 Vaccine-related myocarditis: a pooled analysis of 468 patients. J Magn Reson Imaging.

[20] Patone M (2022). Risk of myocarditis after sequential doses of COVID-19 vaccine and SARS-CoV-2 infection by age and sex. Circulation.

[21] Nakahara T (2023). Assessment of mocardial 18F-FDG uptake at PET/CT in asymptomatic SARS-CoV-2–vaccinated and nonvaccinated patients. Radiology.

[22] Voleti N, Reddy SP, Ssentongo P (2022). Myocarditis in SARS-CoV-2 infection vs. COVID-19 vaccination: a systematic review and meta-analysis. Front Cardiovasc Med.

[23] Bluemke DA (2023). COVID-19 vaccines and myocardial injury. Radiology.

[24] Caobelli F (2023). Cardiovascular magnetic resonance (CMR) and positron emission tomography (PET) imaging in the diagnosis and follow-up of patients with acute myocarditis and chronic inflammatory cardiomyopathy. Int J Cardiovasc Imaging.

[25] Luetkens JA (2019). Comparison of original and 2018 Lake Louise criteria for diagnosis of acute myocarditis: Results of a validation cohort. Radiol Cardiothoracic Imaging.

[26] El Sahly HM (2021). Efficacy of the mRNA-1273 SARS-CoV-2 vaccine at completion of blinded phase. N Engl J Med.

[27] Thomas SJ (2021). Safety and efficacy of the BNT162b2 mRNA Covid-19 vaccine through 6 months. N Engl J Med.

